# Impact of a Workplace Health Promotion Program on Employees’ Blood Pressure in a Public University

**DOI:** 10.1371/journal.pone.0148307

**Published:** 2016-02-03

**Authors:** J. Y. Eng, F. M. Moy, A. Bulgiba

**Affiliations:** Julius Centre University of Malaya, Department of Social and Preventive Medicine, Faculty of Medicine, University of Malaya, Kuala Lumpur, Malaysia; Graduate School of Medicine, Osaka University, JAPAN

## Abstract

**Introduction:**

Workplace health promotion is important in the prevention of non-communicable diseases among employees. Previous workplace health programs have shown benefits such as lowered disease prevalence, reduced medical costs and improved productivity. This study aims to evaluate the impact of a 6-year workplace health promotion program on employees’ blood pressure in a public university.

**Methods:**

In this prospective cohort study, we included 1,365 employees enrolled in the university’s workplace health promotion program, a program conducted since 2008 and using data from the 2008–2013 follow-up period. Participants were permanent employees aged 35 years and above, with at least one follow up measurements and no change in antihypertensive medication during the study period. Baseline socio-demographic information was collected using a questionnaire while anthropometry measurements and resting blood pressure were collected during annual health screening. Changes in blood pressure over time were analyzed using a linear mixed model.

**Results:**

The systolic blood pressure in the hypertension subgroup decreased 2.36 mmHg per year (p<0.0001). There was also significant improvement in systolic blood pressure among the participants who were at risk of hypertension (-0.75 mmHg, p<0.001). The diastolic blood pressure among the hypertensive and at risk subgroups improved 1.76 mmHg/year (p<0.001) and 0.56 mmHg/year (p<0.001), respectively. However, there was no change in both systolic and diastolic blood pressure among participants in the healthy subgroup over the 6-year period.

**Conclusion:**

This study shows that continuing participation in workplace health promotion program has the potential to improve blood pressure levels among employees.

## Introduction

Unhealthy working adults contribute to a substantial economic burden of health-related productivity loss [1, 2]. According to the World Health Organization, non-communicable diseases are estimated to have reduced the Gross Domestic Product by one percent in most low and middle income countries by 2015 [3]. Workplace health programs have been discussed frequently in recent years as a means to protect health and improve productivity among employees [4]. Many workplace health programs have been shown to offer benefits such as reduced sickness absence [5, 6], reduced medical costs [5, 7], improved productivity [1, 8], produced happier, healthier and more loyal employees [9] and lowered disease prevalence [10, 11]. Most of the above were reported from developed countries. There is a scarcity of workplace health programs being reported in the low and middle-income countries such as Malaysia.

Hypertension is a major public health problem in Malaysia [12]. According to the National Health and Morbidity Survey in 2011, an estimated 5.8 million adults (32.7%) in Malaysia were living with hypertension [13]. The high prevalence of hypertension contributed to increased health care expenditure and reduced productivity in Malaysia [14, 15]. Non-modifiable factors such as age, gender and genetics as well as lifestyle factors such as diet, physical inactivity, smoking and alcohol intake are known to be associated with the development of hypertension. Lifestyle factors can be modified to prevent or delay the onset of hypertension. Most workplace health programs which involve lifestyle modification such as physical activity, diet and stress management have been shown to improve employees’ blood pressure [16, 17].

In Malaysia, there have been a few workplace health programs targeting health-related behaviours such as smoking, diet and exercise. Overall, these programs seemed to improve health and reduce modifiable risk factors among employees. For example, results from the Smoking Cessation Program reported that 16.3% of their participants successfully quit smoking [18]. Another health intervention program focused on serum cholesterol among male security guards resulted in a significant decrease in total cholesterol after a 2-year follow-up [19] while a 4-month weight management intervention plan which included aspects of diet, exercise, medicine and psychology among obese employees reported a weight reduction between 4.0 to 17.8 kg [20]. However, these programs did not involve many employees (therefore had small sample sizes) and the duration was not more than two years. In addition, the magnitude of the results varied due to different study designs, populations, types of intervention and outcome measures used. Although the prevalence of hypertension is high among working adults in Malaysia [21], few workplace health promotion programs focused on blood pressure. Our study therefore sought to determine whether a low-intensity workplace health promotion program was able to improve blood pressure among employees. This study may also provide evidence on the health benefits of long-term participation in a workplace health promotion program.

## Methods

### Background

This is a prospective cohort study carried out in a public university from 2008 to 2013. The university was established in 1949, and located in the southwest of Kuala Lumpur, Malaysia. It consists of twelve faculties, two academies and three academic centres. There are about 7,300 employees in the university, the majority (70%) of whom are ethnic Malays. A workplace health promotion program was introduced to all employees aged 35 years and above in 2008. The program aimed to promote employees' health and well-being by increasing health awareness and promoting healthy lifestyle practices. The program was of low intensity and the activities included annual health screening and physical examination, health educational seminars and health exhibitions. All these activities were mainly focused on having a healthy diet, increasing physical activity, quitting smoking and stress management. Besides, employees who found to be at risk of obesity, hypertension, hypercholesterolemia and diabetes were notified and followed up with face-to-face lifestyle counselling conducted by dietitians, or referral for medical treatment when necessary. The first lifestyle counseling session involved baseline assessment of current eating habits, medical history and physical activity followed by suggestions for lifestyle and diet improvement. One session will usually last between 45–60 minutes. Participants were followed up with subsequent counseling session every 6 months to review their progress with each session taking 30 minutes. Education materials such as health brochures were given to the participants at the end of the session. All activities were held in the campus during working hours to encourage participants’ attendance. Generally, programs that only provide medical assessment, behavioural counseling, web-based health programs and/or a less frequent follow-up are considered as low intensity [22] while programs that integrate behavioural education, training courses, health coaches, and social support groups with frequent follow-up are categorized as high intensity [2, 23].

### Study population

All employees aged 35 years and older were invited to participate in the annual health screening and physical examination. Participation in this program was entirely voluntary. The study sample consisted of 1,365 employees who participated in the workplace health promotion program and completed at least one follow-up screening during the 6-year study period (2008–2013). Employees who reported antihypertensive medication changes during the study period were excluded (n = 102).

### Ethics approval

Ethics clearance was obtained from the Medical Ethics Committee of the Medical Centre (Reference Number: MEC 782.18) and approval was obtained from the management of the university. Written informed consent was obtained from participants before data was collected.

### Data collection

A self-administered questionnaire was used to assess socio-demographic characteristics, medical history, medication use and smoking status. Participants’ occupations were categorized into Academic (which included lecturers, professors and post-doctoral staff), Support I (which included executives and officers) or Support II (which included administrative staff, technicians and general workers). Weight was measured using a SECA portable digital weighing scale (SECA 813, Hamburg, Germany) accurate to 0.1 kg, and height was measured using a SECA stadiometer (SECA 217, Humburg, Germany) accurate to 0.1 cm. Body mass index (BMI) was calculated using the formula weight (kg) divided by height^2^ (m^2^). Smoking habits and history of smoking were evaluated by self-reported tobacco-use questions. Smoking status was classified into three categories: never smoked, former smokers and current smokers. Employees who had never smoked were classified as never smoked, whereas employees who smoked ≥1 cigarettes per day were considered as current smoker, and those who stopped smoking before the baseline examination were considered as former smoker.

Resting blood pressure was measured using a clinically validated oscillometric blood pressure monitor (Omron HEM-907, Japan). Participants were required to rest for 5 to 10 minutes before having their blood pressure taken. The participants were examined in a seated position with the arm placed at heart level. A single blood pressure measurement was taken for each participant. According to the World Health Organization (WHO) [24] and Seventh Report of the Joint National Committee on Prevention, Detection, Evaluation and Treatment of High Blood Pressure (JNC 7) [25], normal blood pressure among adults is defined as a systolic blood pressure (SBP) <120 mmHg and diastolic blood pressure (DBP) <80 mmHg. Participants were grouped into three subgroups according to their hypertension history and baseline blood pressure as per WHO and JNC 7 recommendations:

1. Hypertension—self-reported clinical diagnosis of hypertension, with or without medication use;
2. At-risk—no self-reported medical history of hypertension, yet SBP ≥120mmHg and/or DBP ≥80mmHg at baseline; and
3. Healthy—no self-reported medical history of hypertension and had SBP <120 mmHg and DBP <80 mmHg at baseline [26].

### Statistical Analyses

The 6-year data sets were merged into a common data set and cleaned before analysis. Data were analyzed using SPSS for Window version 21.0. Significant level was pre-set at p < 0.05. The primary outcome measures were employees’ systolic and diastolic blood pressure. Participants who did not have any follow-up measurement or those who had just been prescribed medication for hypertension or had a change in antihypertensive medication during the study period were excluded from the analysis as a prescription of antihypertensive medication or change in antihypertensive medication is likely to improve the participants’ blood pressure.

For baseline analysis, association between categorical variables were tested using the chi-square test while the Mann-Whitney *U* test was used for continuous variables. Linear mixed model analysis was used to examine the change in blood pressure within the participants over the follow-up period. Linear mixed model analysis was selected over conventional methods such as repeated measure ANOVA as linear mixed model was not affected by incomplete observations or missing data and allowed for the modelling of time-dependent change in the variables. As repeated measures of blood pressure tend to be correlated, linear mixed model considers the correlation and combines both random and fixed effects. We used compound symmetry as parameters of the random intercept. The analyses were stratified by hypertension status at baseline as we expected differences in blood pressure change to occur among these subgroups. Age at baseline, gender, ethnicity, occupation, body mass index, smoking at baseline and antihypertensive medication were adjusted in the analyses.

## Results

A total of 1, 365 employees (44.2% men, 55.8% women) were included in the analyses (Table 1). The mean (standard deviation) age of the participants was 46.2 (5.8) years. Most participants were Malays (78.8%) and one third worked as support staff group II. At baseline, 76.9% of the participants reported as never smoked, 10.8% as current smoker and 12.3% as former smoker. Their mean baseline systolic and diastolic blood pressure was 128.6 mmHg and 80.1 mmHg, respectively. About 25% of the participants had healthy blood pressure; 56.7% were at risk of hypertension and 18.2% were hypertensive. Participants with normal blood pressure at baseline were younger compared to those with higher blood pressure. About 83.4% of men and 68.2% of women had SBP ≥120 mmHg and/or DBP ≥ 80 mmHg at baseline. Overall, Chinese had the lowest prevalence of hypertension (9%), followed by other minor ethnic groups (10%), with Malays (19%) and Indians (23%) having the highest prevalence. Participants from the lower socio-economic status (support group II) were more likely to have hypertension, while non-smokers and those with healthy body weight were less prone to hypertension.

**Table 1:** Demographic and clinical characteristics at baseline.

Characteristics	Total (n = 1365)	Healthy^a^ (n = 342)	At risk^b^ (n = 774)	Hyper-tension^c^ (n = 249)	p value*
No. with characteristics (%)					
Gender					
Male	603	100 (16.6)	369 (61.2)	134 (22.2)	<0.001
Female	762	242 (31.8)	405 (53.1)	115 (15.1)	
Ethnicity					
Malay	1075	240 (22.3)	633 (58.9)	202 (18.8)	
Chinese	112	53 (47.3)	49 (43.8)	10 (8.9)	<0.001
Indian	148	40 (27.0)	74 (50.0)	34 (23.0)	
Others	30	9 (30.0)	18 (60.0)	3 (10.0)	
Occupation					
Academic	342	129 (37.7)	174 (50.9)	39 (11.4)	<0.001
Support I	179	46 (25.7)	117 (65.4)	16 (8.9)	
Support II	467	110 (23.6)	279 (59.7)	78 (16.7)	
Smoking					
Current smoker	147	25 (7.3)	94 (63.9)	28 (19.0)	<0.001
Former smoker	168	25 (14.9)	95 (56.5)	48 (28.6)	
Never smoke	1050	292 (27.8)	585 (55.7)	173 (16.5)	
Mean characteristics (±SD)					
Mean age, y	46.2 ± 5.8	44.5 ± 5.4	46.0 ±5.5	49.0 ± 5.7	<0.001
BMI, kg/m^2^	26.7 ± 4.5	24.7 ± 4.3	26.8 ±4.3	28.8 ± 4.7	<0.001
Blood pressure					
Systolic, mmHg	128.6±18.0	108.9±7.7	132.0±12.6	144.3±18.9	<0.001
Diastolic, mmHg	80.1±11.2	68.5±6.4	82.4 ±8.2	88.9±12.1	<0.001

^a^No self-reported medical history of hypertension and had a measured systolic <120 mmHg and diastolic <80 mmHg at baseline.

^b^No self-reported medical history of hypertension and had a measured systolic ≥120 mmHg and/or diastolic ≥80 mmHg at baseline.

^c^ self-reported clinical diagnosis of hypertension at baseline.

* Chi-square test for categorical variables; Mann-Whitney *U* test for continuous variables.

Table 2 presents the results of linear mixed model on SBP adjusted for age, gender, ethnicity, occupation, smoking, antihypertensive medication and BMI. Overall, no significant change was observed in SBP over 6 years after adjusting for confounders. However, in the stratified analysis according to baseline hypertension risk, participants in the hypertension subgroup showed the greatest improvement in SBP (-2.36 mmHg per year, p<0.0001). There was also significant improvement in SBP among those at risk of hypertension, but this was of smaller magnitude (-0.75 mmHg per year, p<0.001). Although there was an increase in SBP among participants in the healthy subgroup (1.89 mmHg per year, p< 0.001), the mean SBP was still within the normal range. Overall, the SBP among men was higher than women (6.45 mmHg, p<0.001). On average, every one-year increase in age was associated with a 0.43 mmHg increase in SBP. The reduction in SBP among participants with lower occupational status (Support II) was significantly greater (-3.26 mmHg, p<0.001) than those with higher occupational status (Academic). Compared to those who had never smoked, smokers demonstrated a larger reduction of 4.79 mmHg (p<0.001) in SBP, while there was no significant reduction among former smokers (0.54 mmHg, p>0.05). The SBP among those taking antihypertensive medication was 9.47 mmHg (p<0.001) greater than the non-medication group. For every unit increase in Body Mass Index, there was an increase of 0.55 mmHg (p<0.001) in SBP.

**Table 2:** General linear mixed model estimates for systolic blood pressure over 6-year.

Effects	All	Healthy^a^	At risk^b^	Hypertension^c^
	(n = 1365)	(n = 342)	(n = 774)	(n = 249)
**Fixed Effects**				
Intercept	88.78 (4.18)ƚ	101.54 (5.87)ƚ	97.36 (4.72)ƚ	126.34 (15.35)ƚ
Time	-0.10 (0.13)	1.89 (0.22)ƚ	-0.75 (1.56)ƚ	-2.36 (0.56)ƚ
Age at baseline	0.43 (0.07)ƚ	-0.08 (0.09)	0.44 (0.07)ƚ	0.22 (0.23)
Gender (Male)	6.45 (0.92)ƚ	4.63 (1.30)ƚ	4.93 (1.02)ƚ	1.56 (3.22)
Ethnicity				
Malay	1.79 (2.27)	4.16 (3.06)	1.98 (2.47)	0.66 (8.97)
Indian	-1.22 (2.48)	1.14 (3.24)	0.56 (2.78)	-0.32 (9.58)
Others	-3.05 (2.50)	-0.12 (3.27)	-0.45 (2.82)	-4.47 (10.24)
Chinese	Reference	Reference	Reference	Reference
Occupation at baseline				
Academic	Reference	Reference	Reference	Reference
Support I	-0.04 (0.96)	-1.75 (1.27)	0.59 (1.04)	-3.30 (3.80)
Support II	-3.26 (0.86)ƚ	-1.49 (1.05)	-1.86 (1.02)	-3.32 (3.37)
Smoking at baseline				
Current smoker	-4.79 (1.43)*	-3.76 (2.21)	-5.17 (1.53)*	-2.98 (4.63)
Former smoker	-0.54 (1.29)	-0.59 (2.02)	-0.81 (1.44)	0.16 (3.70)
Never smoked	Reference	Reference	Reference	Reference
Hypertensive medication	9.47 (0.07)ƚ	-	-	-0.17 (2.89)
BMI (kg/m^2^)	0.55 (0.08)ƚ	0.18 (0.17)*	0.39 (0.08)ƚ	0.34 (0.27)
**Random Effects**				
Covariance of Random	76.88 (5.47)	21.39 (4.81)	51.59 (5.32)	111.50 (23.88)
Intercept and Slope				
Variance of measurement error (residuals)	131.43 (3.70)	111.43 (5.88)	116.67 (4.19)	202.98 (17.11)

Adjusted for age, gender, ethnic, occupation, smoking, hypertensive medication and BMI.

The data represents Estimate (Standard error of mean).

Note:

^ƚ^ p-value <0.001;

*p-value <0.05.

^a^No self-reported medical history of hypertension and had a measured systolic <120 mmHg and diastolic <80 mmHg at baseline.

^b^No self-reported medical history of hypertension and had a measured systolic ≥120 mmHg and/or diastolic ≥80 mmHg at baseline.

^c^ self-reported clinical diagnosis of hypertension at baseline.

As presented in Table 3, there was a significant reduction in DBP over the study period (-0.22 mmHg per year, p<0.05). The reduction in DBP among the hypertensive subgroup (-1.76 mmHg per year, p<0.001) was larger than those in the at risk subgroup (-0.56 mmHg per year, p<0.001). However, there was a significant increase in DBP among the participants in the healthy subgroup (0.88 mmHg per year, p<0.001). Men had higher DBP than women (3.27 mmHg, p<0.001). Participants who took antihypertensive medication had an increase in DBP over the study period (5.55 mmHg, p<0.001). The average increase in DBP was 0.61 mmHg (p<0.001) for every unit increase in BMI. Significant increase was observed among the at risk and healthy subgroups, but not in the hypertensive subgroup.

**Table 3:** General linear mixed model estimates for diastolic blood pressure over 6-year.

Effects	All	Healthy^a^	At risk^b^	Hypertension^c^
	(n = 1365)	(n = 342)	(n = 774)	(n = 249)
**Fixed Effects**				
Intercept	57.03 (2.82)ƚ	55.46 (4.48)ƚ	86.26 (3.27)ƚ	87.64 (9.40)ƚ
Time	-0.22 (0.09)*	0.88 (0.17)ƚ	-0.56 (0.10)ƚ	-1.76 (0.35)ƚ
Age at baseline	-0.06 (0.04)	-0.02 (0.07)	-0.03 (0.05)	-0.12 (0.14)
Gender (Male)	3.27 (0.61)ƚ	2.73 (0.98)*	1.70 (0.70)*	2.61(1.95)
Ethnicity				
Malay	1.77 (1.51)	2.77 (2.31)	0.56 (1.11)	1.40 (5.45)
Chinese	Reference	Reference	Reference	Reference
Indian	1.05 (1.66)	2.52 (2.46)	2.26 (1.90)	0.28 (5.81)
Others	0.13 (1.67)	1.71 (2.49)	1.71 (1.94)	-1.12 (6.20)
Occupation at baseline				
Academic	Reference	Reference	Reference	Reference
Support I	1.24 (0.64)	0.80 (0.96)	1.13 (0.70)	0.24 (2.04)
Support II	-0.85 (0.58)	-0.05 (0.79)	0.25 (0.70)	-0.82 (2.04)
Smoking at baseline				
Current smoker	-1.80 (0.95)	-1.82 (1.67)	-1.67 (1.05)	-2.61 (2.79)
Former smoker	0.22 (0.86)	-0.73 (1.53)	0.34 (0.99)	-0.13 (2.24)
Never smoke	Reference	Reference	Reference	Reference
Hypertensive medication	5.55 (0.77)ƚ	-	-	0.54 (1.76)
BMI (kg/m^2^)	0.61 (0.05)ƚ	0.46 (0.08)ƚ	0.52 (0.06)ƚ	0.25 (0.17)
**Random Effects**				
Covariance of Random	34.87 (2.49)	12.01 (2.81)	25.07 (2.47)	39.79 (9.13)
Intercept and Slope				
Variance of measurement error (residuals)	64.88 (1.73)	64.79 (3.43)	52.18 (1.87)	77.33 (6.62)

Adjusted for age, gender, ethnic, occupation, smoking, hypertensive medication and BMI.

The data represents Estimate (Standard error of mean).

Note:

^ƚ^ p-value <0.001;

*p-value <0.05.

^a^No self-reported medical history of hypertension and had a measured systolic <120 mmHg and diastolic <80 mmHg at baseline.

^b^No self-reported medical history of hypertension and had a measured systolic ≥120 mmHg and/or diastolic ≥80 mmHg at baseline.

^c^ self-reported clinical diagnosis of hypertension at baseline.

## Discussion

Our participants were from a work cohort of a public university in Kuala Lumpur. The prevalence of self-reported hypertension (diagnosis made by a clinician) among our participants was 18.2%. However, more than half of them (57%) were found to have blood pressure higher than the normal cut-off values. The combination of lifestyle risk factors as well as low level of awareness and control on hypertension have probably contributed to the increased risk of hypertension among employees[12]. Therefore, the existing health promotion program is timely in increasing awareness and educating our employees especially those high risk individuals to practice healthier lifestyle in the prevention of hypertension.

We observed similar characteristics in age and gender among those with hypertension compared to the national statistics, where older participants and men were more likely to have hypertension. Our results showed ethnic Indians had the highest prevalence of hypertension, contradicted with the fourth National Health and Morbidity Survey (NHMS IV) [13]. In NHMS IV, there was comparable prevalence of hypertension among the three main ethnic groups: Malays (34%), Chinese (32%) and Indians (31%) [13]. This discrepancy could be due to the differences in demographic and socio-economic factors. All our participants were adults aged 35 to 60 years working in an urban area, whereas the NHMS IV was a nation-wide population-based survey including a wide range of age and occupations. In addition, the number of ethnic Chinese, Indians and other ethnic groups in our study were not proportional to the national ethnic distribution. Thus, we could not reliably present more precise prevalence estimates for these ethnic groups.

The observed higher prevalence of self-reported hypertension in the lower occupational group was similar to that obtained in other local studies [27, 28]. Among smokers, only 7% reported to have normal blood pressure at baseline, suggesting that smoking contributed to increased blood pressure. The possible underlying mechanism maybe nicotine-induced activation of the sympathetic nervous system [29, 30]. Participants taking antihypertensive medication had higher systolic and diastolic blood pressure indicating insufficient control of blood pressure. Our results demonstrated a positive association between BMI and blood pressure, similar to that reported elsewhere [31, 32].

This study examined the impact of a low-intensity workplace health promotion program on blood pressure among a group of university employees. Overall, SBP improved 0.1 mmHg per year and DBP improved 0.2 mmHg per year. These findings were clinically insignificant when we analysed the data of all participants collectively. However, when we stratified the analysis by subgroups of hypertension, there was a reduction of almost 2 mmHg per year among participants from both hypertension and at risk of hypertension subgroups. This improvement could be contributed by the effectiveness of individual lifestyle behavioural counselling that managed to increase awareness and positive lifestyle behaviours (diet and physical activity) in good control of blood pressure. Our finding is consistent with other studies that demonstrated improvement in blood pressure among participants enrolled in educational lifestyle counselling [33, 34]. Improvement in blood pressure was greater in the hypertension subgroup than the at risk subgroup. This is possibly due to the relative dependence of blood pressure reduction on the baseline blood pressure, meaning those with higher initial blood pressure experienced the greatest reduction. In addition, the lower improvement in blood pressure among the at risk subgroups may be due to the greater increase in their BMI compared to the hypertension subgroup (data not shown). The systolic and diastolic blood pressure among the healthy subgroup increased over time. This could be due to the unavoidable consequence of ageing and less attention was paid to this subgroup as lifestyle counselling was only given to those at risk. The small magnitude of improvement in blood pressure for all participants may be diluted by the results from the healthy subgroup. Although the overall improvement in our study is unlikely to be clinically significant, we believe these results are important because this workplace health promotion program was able to identify at-risk employees and provided access to health promotion activities to improve health.

Some risk factors for hypertension such as age, gender, smoking and body mass index have been extensively documented [35, 36]. Generally, our findings are similar to previous studies. Some [37, 38] but not all [39, 40] studies suggested that smoking contributes to elevated blood pressure. Studies suggested that age, body weight, years of smoking and number of cigarettes may affect blood pressure among smokers [41]. In our study, the reduction in SBP was greater among smokers than those who had never smoked. Earlier studies suggested that lower blood pressure among smokers could be due to the chronic effect of cotinine on vascular smooth muscle fibres [42], or the nicotine sympathetic pressor effect [42, 43]. Participants with lower occupational status experienced greater improvement in SBP than high occupational status. The participants in high occupational status were mainly academic staff and this occupation group was reported to have higher levels of stress [44, 45]. A previous study suggested that management of blood pressure may be particularly difficult among employees with high stress [46]. Surprisingly, antihypertensive medication did not manage to control the blood pressure among hypertensive participants. This could be due to their inadequate compliance to medication in terms of dosage or frequency; practice of healthy diet (less salt less fat etc) and physical activity. Similar findings were observed in a community-based lifestyle intervention study [47].

Many studies have reported on the effect of workplace health programs on blood pressure and/or healthcare expenditure for hypertension treatment. A physical activity web-based program among employees in a university campus in Spain showed a significant improvement in both systolic and diastolic levels [48]. In Mexico, a workplace health program targeting employees with hypertension in a public university was found to reduce annual healthcare costs by USD5.3 with each dollar invested in the physical activity intervention program [49]. A three-month intervention healthy lifestyle program among a group of blue-collar employees demonstrated an average before-after improvement in systolic (Δ = -6±11 mmHg) and diastolic blood pressure (Δ = -4±7 mmHg) [50]. The results from a randomized controlled trial among a group of white-collar employees showed that a 4-month healthy diet and physical activity program improved the systolic and diastolic blood pressure by 1.4 mmHg and 2.9 mmHg, respectively [51]. Repeat participation in workplace health promotion programs have been shown to prevent high blood pressure [52]. Workplace health promotion programs seem to offer an opportunity for early detection of hypertension and are believed to reduce the disease burden among employees. Due to the heterogeneity in study population, study designs, intervention characteristics and outcome of study, it is difficult to make comparisons across studies [2, 53].

The clinically insignificant reduction in blood pressure achieved in our study may be explained by the low intensity level of the health promotion program. Although there is no conclusive correlation between program intensity and impact [54]; results from a meta-analysis suggests that higher intensity programs leads to better results [53]. The overall improvement in blood pressure in our study may be diluted by the subgroup with no medical history of hypertension and normal blood pressure at baseline. However, our results emphasized the importance of workplace health promotion programs through screening, awareness raising and education on prevention of hypertension.

There are some limitations which need to be addressed. Participation in this program was voluntary which may introduce selection bias. In addition, participants with at least one follow-up visit were included. It is reasonable to expect these participants to be more health conscious [55]. The low number of Chinese, Indians and other ethnic groups may not provide reliable or precise estimates for these ethnicities. Therefore, our results may only be generalized to working population in the public sector and best interpreted for the Malay ethnic group. Due to the logistic constraints during data collection, blood pressure was only measured once and lifestyle factors such as physical activity and diet were not enquired. Although multiple blood pressure observations is much better than a single observation, some studies reported that the difference in SBP (1.0–5.0 mmHg) and DBP (1.5–2.0 mmHg) between two observations taken at intervals of 30 seconds to 2 minute was negligible [56, 57].

Despite the limitations mentioned above, this study has a few strengths which warrant discussion. Although missing data is a common limitation in all follow-up studies including ours, we addressed this limitation by using a linear mixed model analysis that accounted for incomplete data. Linear mixed model uses all available data instead of excluding participants with missing data points or require the use of data imputation which might contribute to bias. This health promotion program with its six-year follow up period reflected its sustainability and enabled the comparison of subgroups and examination of blood pressure changes over time. This may be one of the longest running workplace health promotion programs in our country.

There are many challenges in implementing workplace health promotion programs and we would like to make the following recommendations based on our experience. First, the design of the programs should focus on primary prevention that addresses health-related behaviours and risk factors. In addition, the education should not only focus on employees who are at risk, but also the healthy ones. As they age, their health will deteriorate if they do not take any action. Second, achieving adequate participation as well as engagement of employees in the program is essential to sustain the program and to ensure good response rate in all activities over time. High intensity intervention programs with multi-component activities such as diet intervention, physical activity programmes and frequent follow-up consultation are suggested to encourage continuing participation and improve employees’ health. Finally, future research should measure the cost effectiveness of the program and to explore the factors affecting behavioural changes more in-depth using a qualitative approach.

## Conclusion

The results of this study showed some improvement in blood pressure among employees who participated in a low-intensity workplace health promotion program. This suggests that repeat engagement in long-term workplace health promotion program provides an opportune setting to reduce hypertension risk among employees. Activities such as periodic health screening can serve as the first step to identify employees who are at risk while health promotion and intervention programs can help to increase awareness and encourage adoption of healthy lifestyles among employees. Further studies may also be conducted to examine the cost-effectiveness of workplace health programs.

## Supporting Information

S1 Data Original data in SPSS. (SAV)
